# Influence of boron- and barium-containing modifiers on the structure of low-chromium cast iron

**DOI:** 10.1016/j.heliyon.2022.e11496

**Published:** 2022-11-10

**Authors:** D.R. Aubakirov, A.Z. Issagulov, A.A. Akberdin, Sv.S. Kvon, V.Yu. Kulikov, S.K. Arinova, A.M. Dostaeva, Ye.P. Chsherbakova, B.B. Sarkenov, A.K. Narembekovа

**Affiliations:** a Karaganda Technical University Named after Abylkas Saginov, Department of Nanotechnology and Metallurgy, Karaganda, Kazakhstan; b Chemical and Metallurgical Institute Named after Zhantore Abishev, Karaganda, Kazakhstan

**Keywords:** Low-chromium cast iron, Modification, Boron, Barium, Wear resistance, Hardness

## Abstract

The paper presents the results of studying the effect of modifying low-chromium hypoeutectic cast iron (chromium content about 1%) with boron- and barium-containing additives on its structure and hardness. The modification was carried out with carbothermicferroboron (0.08% of weight of the liquid metal); ferrosilicobarium (0.05% of weight of liquid metal); complex boron barium ferroalloy (0.14% by weight of liquid metal). The microstructure and properties were compared with a sample of unmodified cast iron of the same composition.

The Thixomet Pro software was used as the main method of the quantitative analysis of the microstructure that allows quantitative metallographic analyzing with high accuracy and repeatability.

The analysis of the results obtained shows a positive effect of modification on changes in the microstructure. In all the experimental samples, grinding and more uniform distribution of carbides, as well as transformation of the carbides morphology from dendritic to compact granular form, take place. Pearlite colonies in the modified cast iron are characterized by a higher degree of dispersion than in the reference sample. Such changes in the structure lead to increasing the hardness of the prototypes*.*

## 1 Introduction

The raw nature of the economy of Kazakhstan determines a large volume of mining, crushing, grinding and dressing the mineral raw materials. The equipment used requires high impact strength and wear resistance. In this industry there are parts cast of the most accessible alloy for domestic manufacturers: white cast iron for grinding media, mill lining elements, beaters of hammer mills, parts of industrial pumps, etc.

The durability of such castings and parts is largely determined by the properties of the alloy grade selected for their manufacturing. At the same time, operating conditions often impose two mutually exclusive requirements on white cast iron castings in terms of special properties: high wear resistance and sufficient toughness, which determines the impact resistance of parts. The economic considerations dictate the need to use relatively inexpensive materials, which requires a reasonable compromise.

At present, in the most advanced industries, a significant increase in the wear resistance of white cast iron castings is usually achieved by using high or complex alloying methods followed by heat treatment, when special high-hard alloying element carbides are formed in the cast iron, and the metal matrix is formed of martensite or alloyed austenite [1, 2, 3]. The other alternative methods of improving the working properties of white cast iron, such as purging with active gases, out-of-furnace treatment of metal with ultrasound, vibration, etc., have not yet received wide practical application due to a number of constraining reasons, among which there is a high cost of equipment, the complexity of use in real production conditions and finally the absence of a single reliable opinion on the effectiveness of using such technologies. Even such a fairly traditional method of improving the structure and properties of the metal as inoculation, is mainly used for graphitizing processing of cast irons with lamellar and nodular graphite, although many researchers are of genuine interest in the features of the modifying effect of various additives on the quality of low-alloyed white cast irons castings [4, 5].

Today, there are more than 500 different types and names of modifying additives for cast irons and steels that can contain from 2 to 15 or more active elements, which is due to the presence of a large number of different theories of modification and out-of-furnace processing of iron-carbon alloys.

The prevailing opinion is that the presence of carbides of the Me_7_C_3_ and or MeC type in the structure of wear-resistant cast irons already provides them with a complex of high performance, and therefore attention of most researchers is focused on studying the features of the structure of high-chromium and complex-alloyed white cast irons, and the modes of the applied heat treatment. A lot of work is being done to study the effect on their structure and properties of various modifying and microalloying elements. However, it must be remembered that in addition to the type of carbides formed, a number of other characteristics, such as the features of the metal base of the alloy, the morphology and dimensions of the carbide phase, are equally important for the wear-resistant properties of cast iron [6]. If the type of carbides formed is mainly affected by the degree of alloying of the alloy, then the size and shape of the structural components can be changed by modifying processing.

In publications [7, 8, 9, 10], the authors confirm the possibility of significant increasing the wear-resistant properties of low-alloy cast irons with carbides of the Me_3_C type due to their grinding and more isolated and uniform distribution in the matrix as a result of modification. In work [11], the possibility of changing the type of carbides in low-chromium cast irons as a result of modifying FeSiMg is thermodynamically substantiated.

The results of study [12] indicate the possibility of increasing the impact strength of chromium cast iron (C 2.5÷3%, Cr 20%) by modifying with strontium, and in work [13] a similar effect was achieved when metal was treated with titanium carbide (TiC up to 1.0% wt).

Much attention of researchers is paid to studying the modifying effect of complex additives containing REM on the structure and properties of white cast iron [14, 15, 16, 17, 18]. Work [15] describes the results of modifying chromium alloys (C 1.5÷4.2%, Cr 14%) with cerium (Ce 0.1÷0.5% wt). The best results in terms of strength and wear resistance were obtained at ∼ 0.2–0.3% Ce, and the maximum hardness (HRC 68.5 units) was in cast iron with the carbon content of 3.5% wt. The authors of studies [16, 17] achieved the grinding of primary and eutectic carbides in chromium cast iron by modifying it with the liquid Fe–Si-Re alloy.

At the same time, the facts of the positive effect of boron additives [19, 20, 21, 22] on the properties of chromium white cast irons are known. Study [23] indicates the positive effect of boron microadditives on the microstructure, mechanical properties, and abrasion resistance of low-chromium cast irons.

The results of works [24, 25] indicate high efficiency of boron in the composition of complex modifiers containing REM: in the complex modification of chromium cast iron with RE-B additives, the morphology of carbides is significantly improved and the metal base is crushed.

There is an interesting fact that there is still no reliable information of the use of barium-containing additives for modifying wear-resistant cast irons in numerous scientific and information bases, although barium has high chemical affinity for nitrogen, sulfur and oxygen and, according to the intensity of its effect on the structure and properties, it is one of the most effective deoxidizers, desulfurizers and modifiers of iron and steel [26].

The conducted literature review reveals that most of the available works describe the effect of barium-containing additives on the structure and properties of steels [26, 27, 28, 29], as well as gray and high-strength cast irons [30, 31, 32].

Classic work [26] is dealing with a review of the various modifiers effect on the structure of cast irons, including low-chromium white cast irons. However, the analysis carried out by the authors shows that almost all the studies dealing with the effect of modification on the structure are limited only to studying the transformation of the graphite shape, while there is practically no information of the combined use of boron and barium as part of complex modifiers.

Meanwhile, study [33] presents the results of laboratory experiments on the smelting and modification of low-chromium white cast iron (C 3.3%, Cr 1.0%, HRC = 49÷50 units) with FSMg9 ferrosilicon magnesium, FB12 ferroboron and FS60Ba22 ferrosilicobarium. Melting was carried out in a Tamman laboratory furnace, portions of modifiers were introduced into a crucible with cast iron overheated to 1500 °C using a steel rod, and the samples were cast by lost foam casting. Portions of modifiers were tested taking into account the residual content in cast iron of Mg ≈ 0.04÷0.06% wt, B ≈ 0.006÷0.02% wt, Ba≈ 0.005÷0.01% wt. Measuring the hardness of cast samples showed increasing the bulk hardness in samples of low-chromium cast iron modified with ferroboron up to 56.5 HRC (with the residual content of B ≈ 0.006%) and ferrosilicobarium (up to 58–60 HRC), with the residual content of Ba ≈0.01%.

Further, the work at studying the effect of boron- and barium-containing modifiers on the structure and properties of low-chromium cast iron was continued in the industrial conditions of the QazCarbon LLP foundry (Karaganda, Republic of Kazakhstan) [34]. The experiments were carried out on ladle modification of low-chromium white cast iron (C 3.3%, Cr 0.7%) with boron- and barium-containing additives of domestic production: ferroboron, ferrosilicobarium and a new complex boron-barium ferroalloy. Melting was carried out in an industrial induction furnace IChT-5.0, portions of modifiers were fed into the metal stream on the chute of the distributing ladle at the temperatures of 1380–1420 °C, the samples were cast by casting into raw sandy-clay molds. Portions of modifiers were tested taking into account the residual content in cast iron B ≈ 0.006 and 0.02%, Ba≈ 0.005 and 0.01%. When using each of these additives, a positive modifying effect was obtained, which is evidenced by improving the mechanical properties: hardness and impact resistance. The microstructure of the samples was studied by optical microscopy and showed refining the microstructure and changing the nature of the distribution of structural components.

An earlier study [35] describes the results of studies of the structure and mechanical properties of low-chromium cast iron modified with boron- and barium-containing modifiers. In this case, the emphasis was placed on the study of the properties of the alloy—resistance to abrasion and cyclic shock-dynamic effects. When analyzing the microstructure, the most general characteristics were obtained and the method of optical metallography was used for this.

This article presents the results of changes in the microstructure of the same objects, but using the scanning electron microscopy method and Thixomet Pro software.

## 2 Experimental part

### 2.1 Research methodology and materials used

Experiments on the smelting of low-chromium cast iron and modification of boron- and barium-containing ferroadditives were carried out on scientific research laboratory "Bor" of Chemical-Metallurgical Institute named after Zh. Abishev (Karaganda, Republic of Kazakhstan).

The object of the study was low-alloy chromium cast iron with a chromium content of up to 1%, which serves as one of the most affordable materials for the production of cast wear-resistant parts in the Republic of Kazakhstan.

Iron smelting was carried out in a resistance furnace in alundum crucibles. The mass of cast iron of one melting was 0.4–0.5 kg. Modification of cast iron was carried out by immersion of portions of the modifier in a crucible with liquid cast iron at a temperature of about 1500 °C. The determination of the number of introduced modifiers was based on information from previous studies in this area [2, 5, 8, 9], which showed the prospects and effectiveness of their use.

To study the effect of the introduced modifiers on the microstructure of low-chromium cast iron, samples were selected and prepared from prototypes with the best hardness, the characteristics and symbols of which are given in Table 1.

**Table 1:** Types and symbols of the studied samples.

№	Sample characteristics	Conditional designation	Hardness HRC, units	Consumption of the introduced modifier, % by weight of cast iron
1	Unmodified low-chromium cast iron	sample 0	49	-
2	Low-chromium cast iron modified FeB_12_	sample 1	56	0,08
3	Low-chromium cast iron modified FeSi_60_Ba_20_	sample 2	59	0,05
4	Low-chromium cast iron modified borbarium modifier	sample 3	57	0,14

The types of modifiers used, their composition and doses, as well as the methods of the processes of iron smelting, modification and sample preparation are described in detail in this article [35].

## 3 Results of the study and discussion

**Figure 1:**
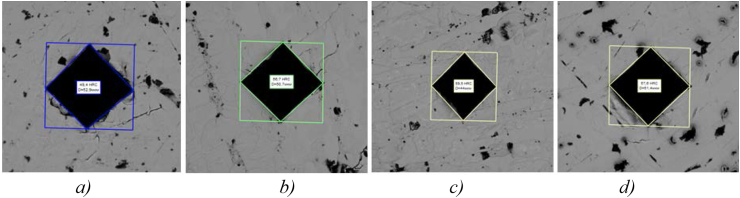
–Measuring hardness of the samples made of low-chromium cast iron (1% Cr): а) sample 0 (49 HRC), b) sample 1 (56 HRC), c) sample 2 (59 HRC), d) sample 3 (57 HRC).

Figure 1 shows the results of measuring the low-chromium cast iron specimens hardness before and after modification that are the closest to the average values.

Figure 1a shows that unmodified low-chromium cast iron (1% Cr), sample 0, has the lowest hardness of 49 HRC, which is quite natural due to the large-lamellar form of the structural components: pearlite and cementite.

**Figure 2:**
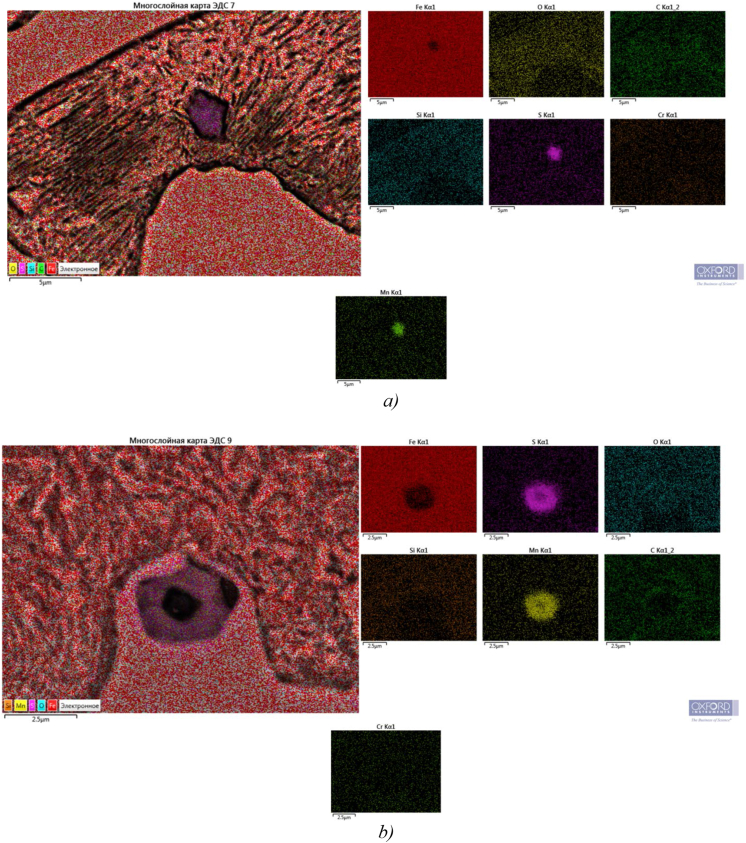
Distribution of chemical elements in sample 2 with finely-dispersed inclusions: а) inside the pearlite colony, b) at the external boundary of the colony.

Sample 2 has the highest hardness (about 59 HRC) (Figure 1c). Barium has a higher affinity for oxygen dissolved in liquid iron than aluminum. Being one of the strongest deoxidizers of steel and cast iron, when introduced into liquid metal, it forms refractory compounds with sulfur and oxygen (t_melt_ BaO = 2113 °C, t_melt_ BaS = 2200°С) [26, 27, 28, 29, 30]. According to the opinion of the authors [26, 30], it can be assumed that the resulting finely dispersed non-metallic inclusions (Figure 2) serve as additional crystallization centers during solidification of the melt and contribute to obtaining a fine-grained dense structure and increased purity of the metal surface.

As can be seen from Figure 2, additional crystallization centers can occur both inside the pearlite colony itself (Figure 2a) and on its outer borders (Figure 2b).

**Figure 3:**
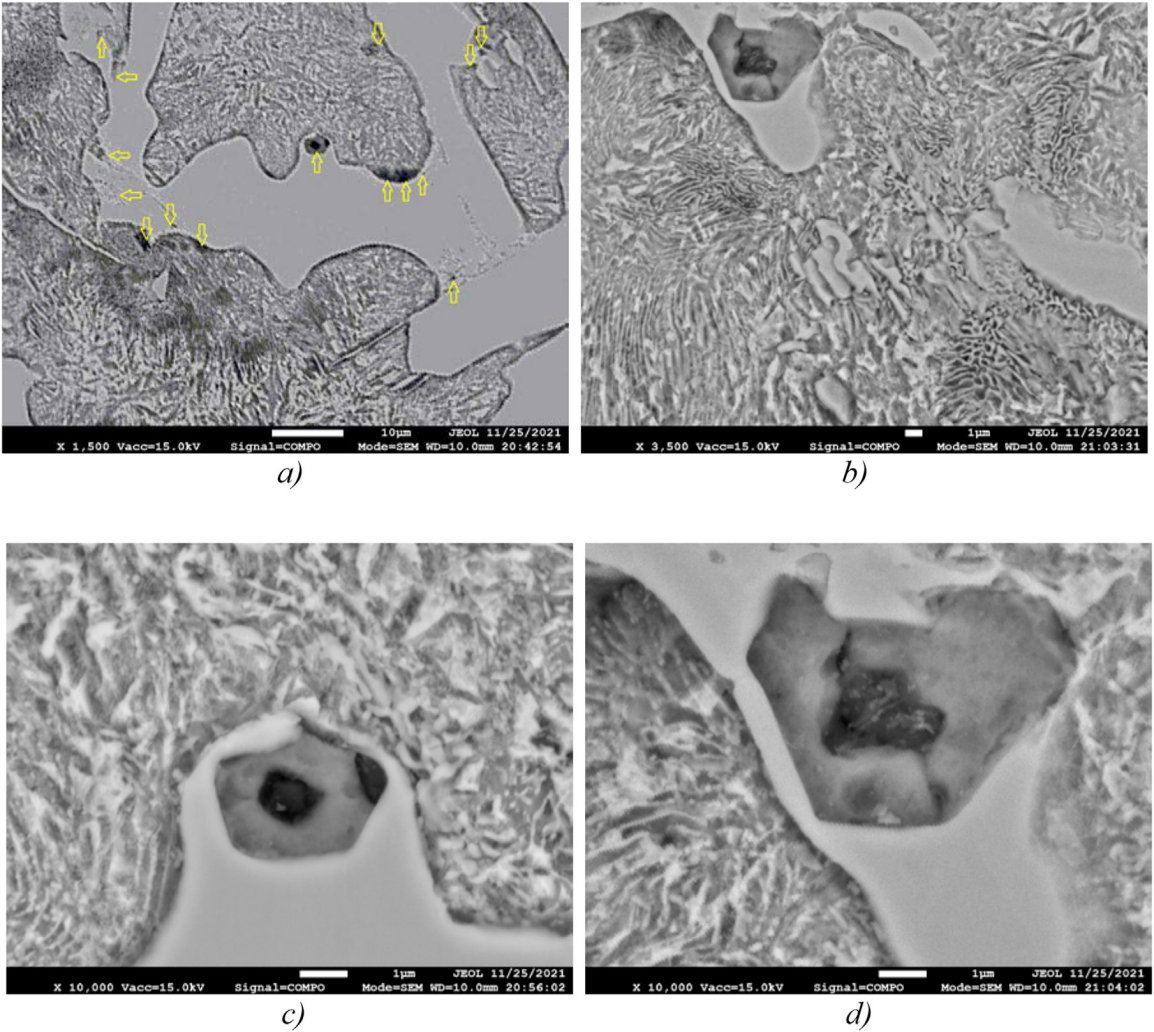
Finely dispersed non-metallic inclusions in the sample 2 structure preventing the growth of the pearlite colony in one direction at different magnifications: a) and b) sulfide inclusions at the boundaries of the growth of pearlite colonies; c) and d) enlarged images of sulfide grains.

The nature of the distribution of fine inclusions limiting the growth of pearlite colonies can be judged from the images in Figure 3a and b, and the isolated arrangement of individual particles is clearly visible in Figure 3c and d.

The authors of [27] suggest that barium atoms prevent the growth of crystallites by condensing mainly ahead of the crystallization front in a thin layer of the liquid phase, which is conditioned by its very low distribution coefficient.

**Figure 4:**
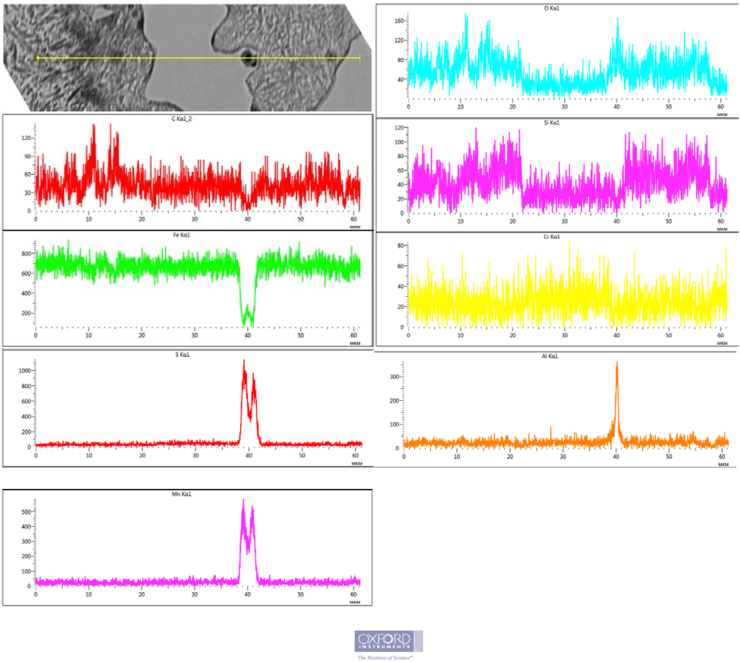
Sulfide inclusion in the phase-boundary space of the sample 2 structure.

Figures 2, 3, and 4 show that there are dispersed sulfide inclusions in the structure of cast iron modified with ferrosilicobarium, which is confirmed by the data of the MRSA analysis. In the spectrogram presented below (Figure 4), it is seen that such an inclusion contains increased concentrations of S, Mn and Al, while the distribution of other elements is quite uniform.

The information analysis carried out [2, 5, 8, 9] suggests that the formation of such fine inclusions is initiated by barium introduced into the modifier, and the resulting fine sulfide inclusions, located both inside and along the boundaries of eutectic colonies, noticeably inhibit their growth, which is one of the main factors in increasing the dispersion and increasing the density of the microstructure of cast iron.

Samples 1 and 3 have almost the same increased hardness of 56–57 HRC (Figure 1, b, d), which confirms the presence of the carbide-stabilizing effect of boron-containing modifiers in cast iron of the experimental composition. It is manifested by increasing precipitation of carbides mainly inside the grains, and not along their boundaries, since boron significantly lowers the surface tension of the grain boundaries, and most of the “active” boron in the solid solution concentrating along the grain boundaries, fills the existing vacancies. This in turn prevents diffusion and reduces carbon segregation along the grain boundaries [20, 21, 22, 23, 24, 25, 26, 27, 28, 29, 30, 31, 32, 33, 34, 35, 36]. Thus, boron atoms in cast iron, being adsorbed on the surface of the solid phase, deactivate graphite nuclei during crystallization and have a stabilizing (deinoculating) effect on the structure.

It can be assumed by analogy with the conclusions of [37], that increasing the hardness of cast iron after the introduction of boron-containing additives is also explained by its microalloying effect with the formation of high-hard boron-containing phases: carboborides of the Fe_3_(C,B) type, as well as the solubility of boron in Cr_7_C_3_ carbides up to 36% (at.), in Cr_3_C_2_ up to 5% (at.)”.

**Figure 5:**
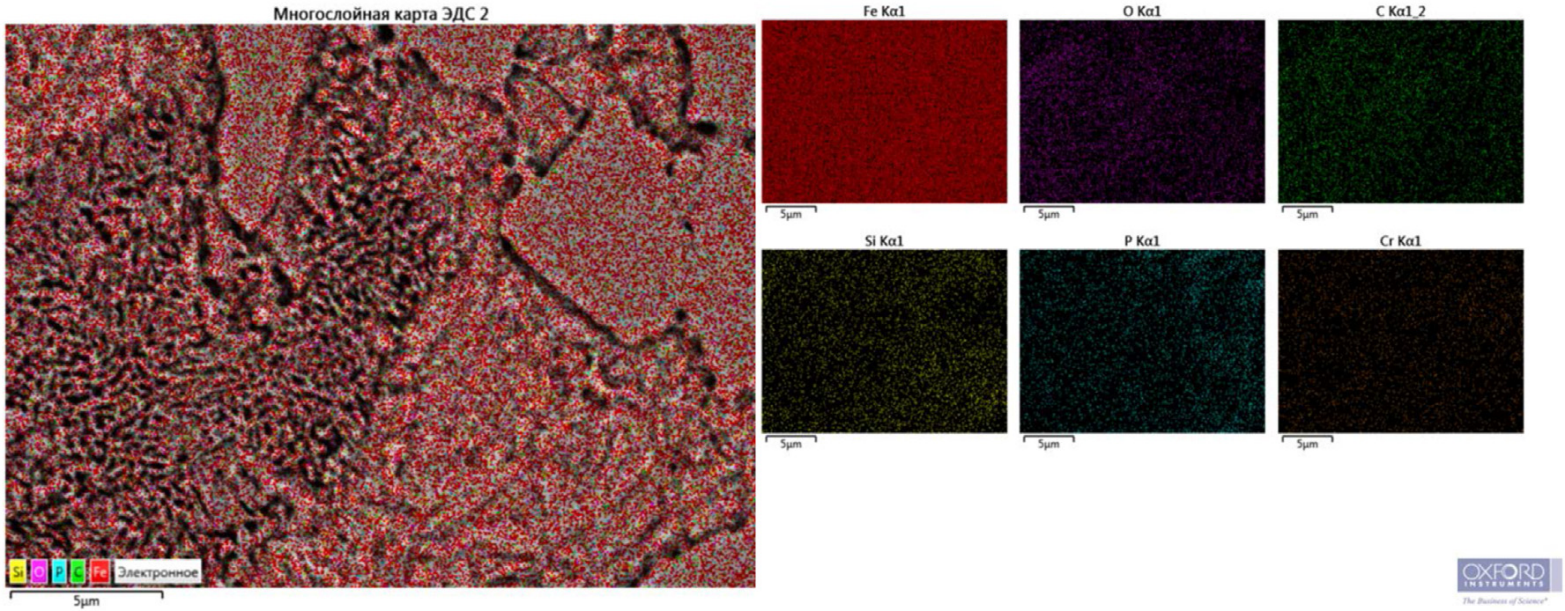
Distribution of chemical elements in the sample 1 structure.

The distribution of elements in the structure of cast iron modified with carbothermalferroboron is quite uniform (Figure 5), without any signs of chemical segregation.

The “clouding” of the alloy by modifier particles leads to significant refinement of the primary grains. Boron prevents releasing excess phases at the grain boundaries, ensures their uniform formation and distribution inside the matrix, improves the state of the grain boundaries, thereby increasing the intergranular strength of cast iron [9, 13].

Boron effects strongly the processes of cast iron crystallization as a surface active element, improves the state of grain boundaries, refines them and additionally deoxidizes the metal, which has a positive effect on the production of castings and their performance properties. Boron also reduces the size of eutectic colonies and transcrystallization in white irons [11].

Boron accelerates the diffusion of chromium in iron, has a high chemical activity towards oxygen and nitrogen dissolved in the metal. The deoxidizing ability of boron is much higher than that of Si, Mn, Cr, V, and the chemical affinity for nitrogen is greater than that of Ca, Cr and V. Boron that actively interacts with sulfur, oxygen and nitrogen in liquid metal, forms new dispersed compounds: sulfides, oxides and nitrides that play the role of embryonic phases during further crystallization. For example, with sulfur, boron forms several sulfides, of which BS, BS_2_, B_2_S_2_ are in the gaseous state, and B_2_S_3_ sulfide is a condensed phase.

The strongest effect of introducing even relatively small doses of boron-containing ferro-additives into cast iron is explained by the complex step-by-step effect of boron: a part of the boron immediately after introducing into the liquid melt is spent for deoxidation and denitrogenation of the metal, and the remaining amount of the so-called "active" boron has a directly modifying effect and microalloys the matrix. Therefore, the nature of the boron effect on the structure and properties of cast iron depends strongly not only on the amount of the additive but also on the initial content of such elements as oxygen and nitrogen in cast iron.

The microalloying effect of boron in chromium cast iron also consists in the condensation of hardening phases FeB and Cr_2_B.

**Figure 6:**
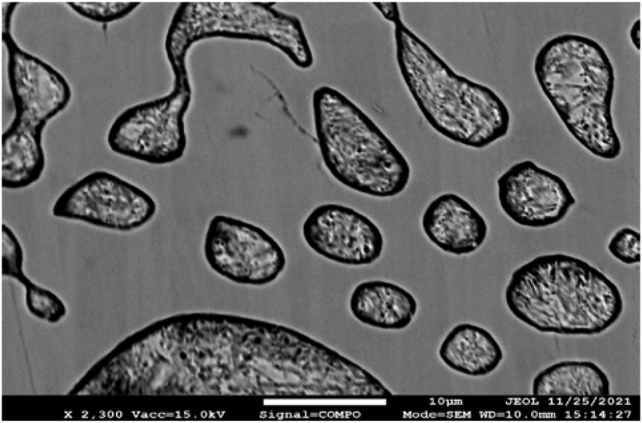
Low-chromium cast iron microstructure modified with FeB12, ×2300.

Figure 6 clearly shows that during crystallization of sample 1, pearlite colonies of a compact spherical or oval shape with clear smooth boundaries were formed. In this case, changing the shape: coalescence and spheroidization of phase precipitates, can also be explained by noticeable changes in the energy ratios at the interphase boundaries due to the appearance of boron at the grain boundaries. Increasing the hardness and wear resistance of cast iron is facilitated, first of all, by changing the morphology of carbides: the transition from the lamellar to the hexagonal shape.

**Figure 7:**
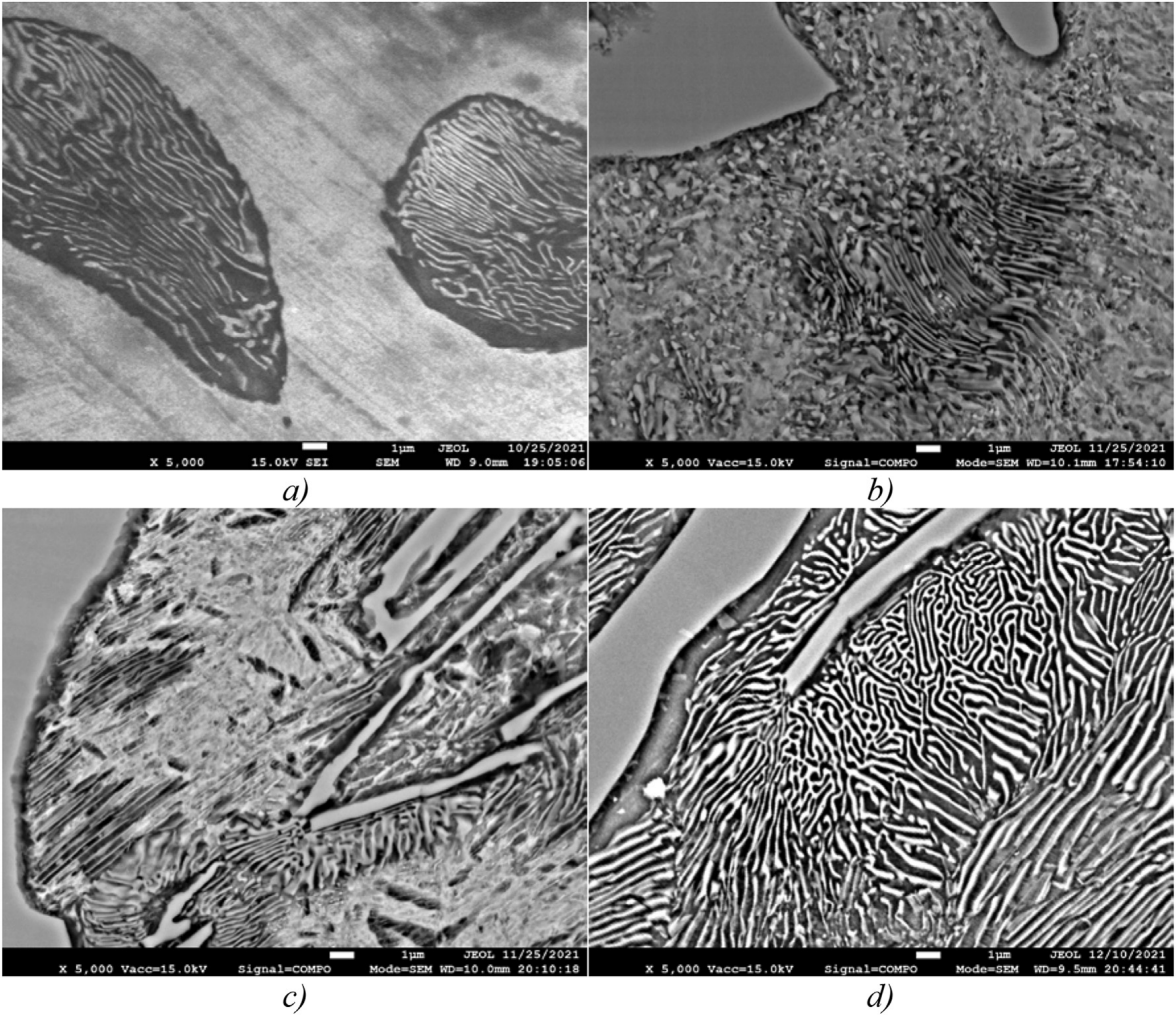
Low-chromium (1% Cr) cast iron pealite structure, ×5000: а) sample 0, b) sample 1, c) sample 2, d) sample 3.

Changing the shape, size and nature of the structural components of low-chromium cast iron distribution before and after inoculation is shown in Figure 7.

Figure 7a shows that in the structure of sample 0, the pearlite structure is lamellar, the ferrite and cementite plates are rather rough and have a large length, which negatively affects the strength properties of cast iron.

In the pearlite structure of sample 1, the ferrite and cementite phases are predominantly in the compact granular form (Figure 7b), which explains its increased impact strength, with the exception of the central region of the colony, where the structural components still retain the lamellar shape.

The structure of sample 2 (Figure 7c) is also characterized by a more compact shape and high dispersion of the components, however, in some places there are rather large cementite needles and coarser pearlite plates are concentrated at the outer boundaries of the colonies, which can be caused by chemical segregation. We assume that this combination can serve as an explanation for the increased hardness and some embrittlement of the alloy.

**Figure 8:**
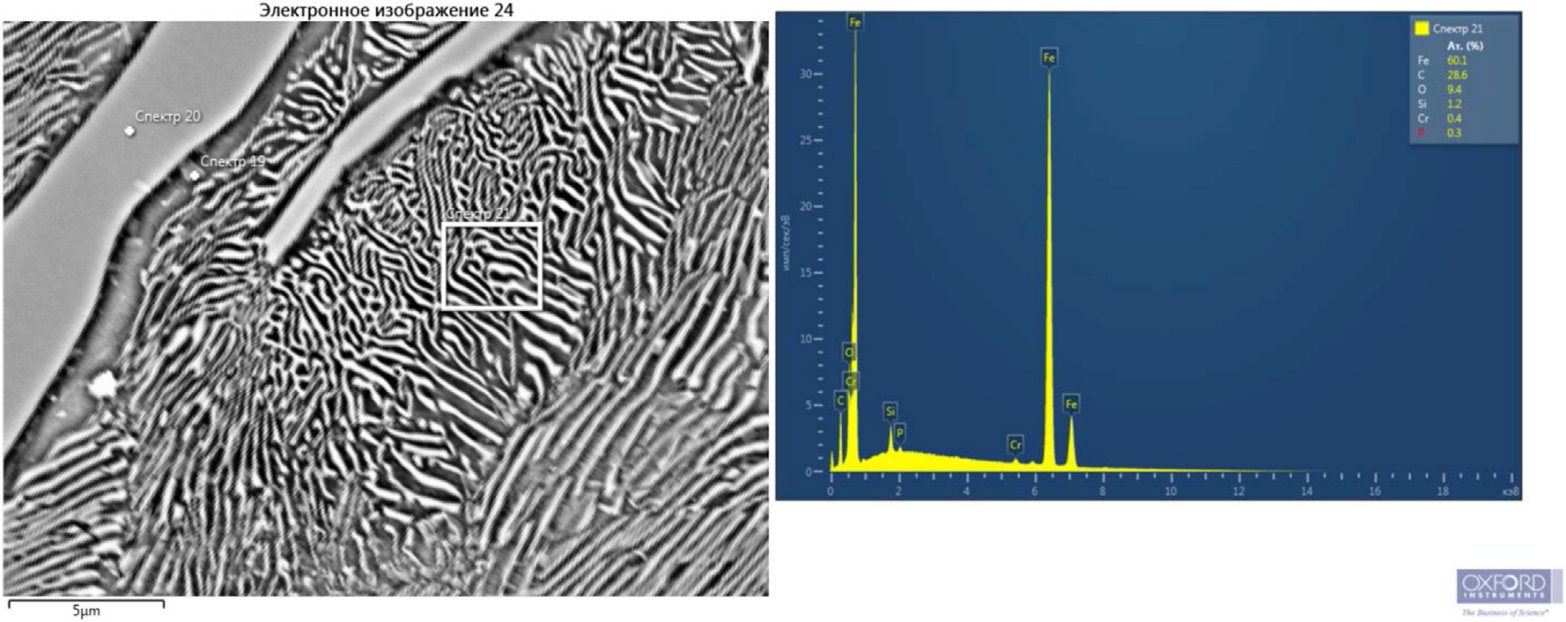
Content of chemical elements in the zone of eutectic colonyin the sample 3 structure.

Sample 3 (Figure 7d) modified simultaneously with both elements, boron and barium, also has a finer structure. We assume that inclusions of the ternary eutectic A + Fe_3_C + Fe_3_P of the lamellar structure are located inside the pearlite colony (Figure 8), which favorably affects the casting and antifriction properties of cast iron. The refinement of the eutectic grain contributes, to some extent, to increasing the strength and toughness of cast iron. Areas of coarse lamellar perlite presumably caused by segregation of elements, are mainly concentrated in the center of the colony.

Due to the high chemical activity, a part of boron that has transited into the metal interacts with oxygen and nitrogen. One of the most effective ways to prevent the binding of boron is introducing boron in the composition of complex boron-containing ferroalloys with a reduced content of boron. It is known that when using this method, the degree of transition of boron into metal increases [38]. Lowering the content of boron in the modifier makes it possible to lower the melting point of the additive and thereby promotes better assimilation of the active components.

It is known that the use of barium as part of complex modifiers significantly increases the duration of their action [31]. As part of the boron-barium modifier, barium reduces the reactivity of boron, thereby increasing its modifying ability. In this case the degree of assimilation of boron in cast iron rises to 75–80% instead of 40–50%, when boron is supplied as part of the standard grades of FeB16-20 ferroboron, and accordingly the modifying effect is prolonged by increasing the duration of the modifier to 25 min or more. When boron is supplied as part of standard FeB16-20 ferroboron grades, its assimilation is 40–50%, and survivability is no more than 5 min from the moment of introducing into the melt.

Boron and barium in the composition of the applied modifiers increase the degree of cast iron supercooling, which contributes to the formation of a large number of small crystals during solidification. We assume that increasing the structural components of cast iron modified with boron- and barium-containing additives dispersion occurs as a result of nucleation of many additional centers of crystallization: refractory oxides, nitrides and sulfides of boron and barium, the number of which grows as the process develops, and their growth becomes noticeably more difficult when the growing crystals collide.

**Figure 9:**
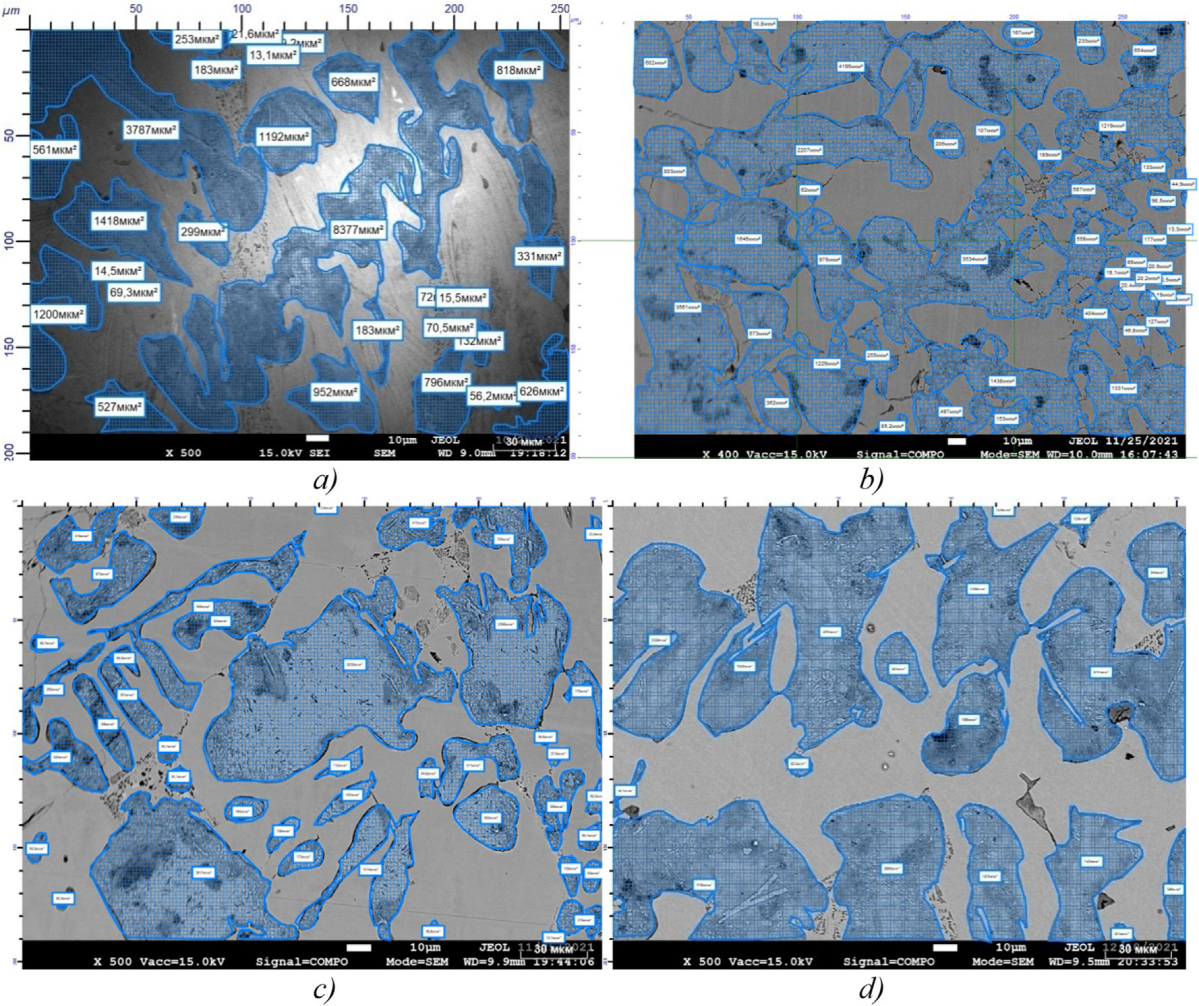
The shape and area of pearlite colonies in low-chromium cast iron, ×500: a) sample 0; b) sample 1; c) sample 2; d) sample 3.

Figure 9 shows the analysis of the qualitative and quantitative characteristics of pearlite colonies of low chromium cast iron that is unmodified and modified with boron- and barium-containing additives using the Thixomet PRO analyzer program.

Figure 9a shows an image of fragmentation of the cast iron microstructure image using the Thixomet analyzer to determine the total area of perlite, as well as the ratio of structural components, Figure 9b, c and d show the same process on samples of modified cast iron.

**Figure 10:**
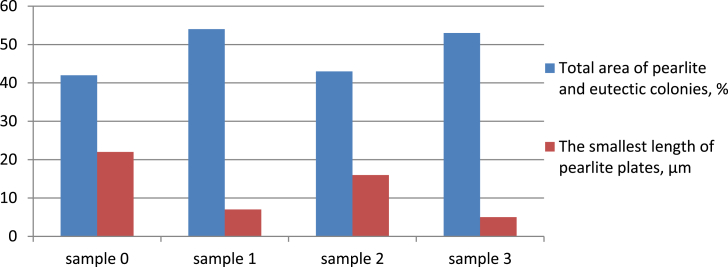
Pearlite amount and the smallest length of pearlite plates in the structure of unmodified and modified cast iron.

The total area of pearlite and eutectic colonies, as well as the minimum lengths of pearlite plates in the structure of unmodified and modified low-chromium cast iron are shown in Figure 10.

Accordingly, the predominance of cementite in the structure of cast iron provides increased hardness and abrasive wear resistance, and increasing the proportion of fine pearlite and eutectic gives increasing the strength and toughness of cast iron.

**Figure 11:**
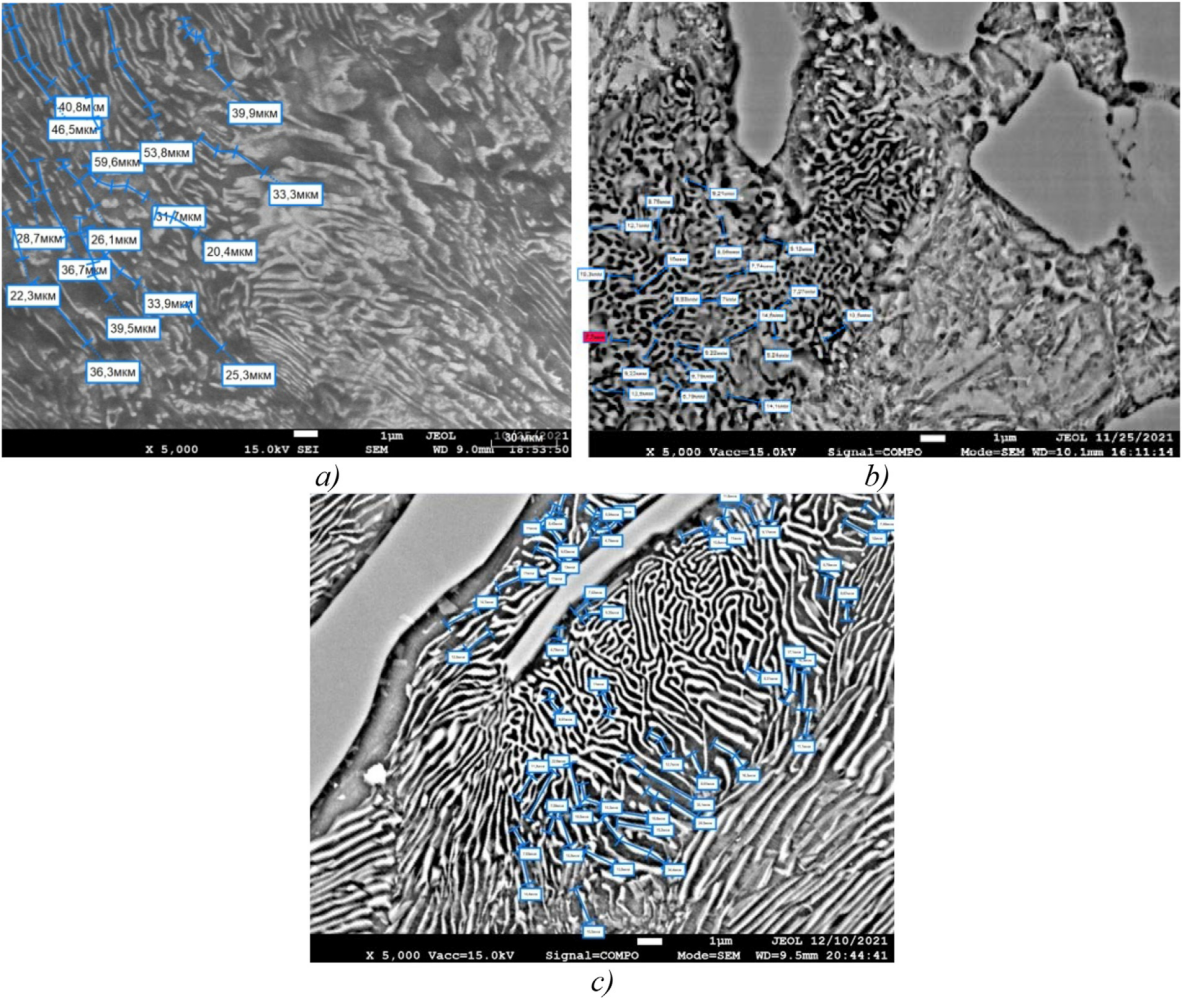
Shape and length of pearlite plates in low chromium cast iron, ×5000: а) sample 0, b) sample 1, c) sample 3.

Figure 11 shows the process of qualitative analysis of the structure of perlite, and more precisely, the measurement of the length of cementite lamellae in perlite.

In the perlite of unmodified cast iron, cementite is formed in the form of elongated branched plates whose length can reach 60 microns or more (Figure 11a), whereas in modified cast iron there is a noticeable grinding of cementite (Figure 11b and c). When using a complex borbarium modifier, grinding of cementite lamellae along the length is observed by more than 4 times (if in unmodified cast iron the smallest length of cementite plates was 22 microns, then after the introduction of the borbarium modifier, this indicator decreased to 5 microns).

## 4 Conclusion

The analysis of metallographic images of unmodified and prototype samples modified with boron- and barium-containing additives shows grinding and more uniform distribution of carbides in the structure of modified cast iron, as well as changing the morphology of carbides from the dendritic to the compact granular form. Pearlite grains in treated cast iron also take on a more compact, finely dispersed shape than in unmodified cast iron of the same composition.

Qualitative analysis of the microstructure of samples of cast iron modified with boron and barium-containing additives showed the presence of numerous dispersed inclusions, mainly of a sulfide nature, both at the phase boundaries and inside pearlite colonies, which, acting as additional crystallization centers, create conditions for the formation of a fine-grained dense structure.

Based on the quantitative analysis of the microstructure of the prototypes, it can be argued that the modification of low-chromium cast iron with a complex borbarium additive contributes to the redistribution of the ratio of structural components in cast iron—the amount of Perlite + Ledeburite increases by 10% (from 80 to 90%), and Cementite decreases from 20 to 10%. At the same time, the grinding of cementite lamellae in perlite occurs by more than 4 times (if in unmodified cast iron the smallest length of cementite plates was 22 microns, then after the introduction of a borbarium modifier, this indicator decreased to 5 microns).

Based on the above, it can be concluded that it is promising to use a complex barbarium additive as a modifier to improve the parameters of the microstructure and, consequently, the working properties of wear-resistant cast iron.

## Declarations

### Author contribution statement

D.R. Aubakirov, Sv.S. Kvon: Conceived and designed the experiments; Analyzed and interpreted the data; Wrote the paper.

A.Z. Issagulov: Conceived and designed the experiments.

А.А. Аkberdin: Conceived and designed the experiments; Analyzed and interpreted the data.

V.Yu. Kulikov: Analyzed and interpreted the data; Wrote the paper.

S.K. Arinova: Performed the experiments; Wrote the paper.

A.M. Dostaeva, Ye.P. Chsherbakova: Performed the experiments; Contributed reagents, materials, analysis tools or data.

B.B. Sarkenov, A.Kh. Narembekovа: Contributed reagents, materials, analysis tools or data.

### Funding statement

This work was supported by the Science Committee of the Ministry of Science and Higher Education of the Republic of Kazakhstan (AP08855477 «Developing and implementing the technology of producing the «Nihard» class cast irons with increased operational properties for mining and processing equipment parts»).

### Data availability statement

The authors are unable or have chosen not to specify which data has been used.

### Declaration of interests statement

The authors declare no conflict of interest.

### Additional information

No additional information is available for this paper.
